# Supplementary material for: Chromothripsis during telomere crisis is independent of NHEJ, and consistent with a replicative origin
Source: Genome Res. 2019 May;29(5):737–49. doi: 10.1101/gr.240705.118 (PMC6499312; doi:10.1101/gr.240705.118)
Supplement: Supplemental Material [file supp_gr.240705.118_Supplemental_file_1.zip › contigs/annotated_contigs/DB69/contig.2.DB69_length_356_mean_cov_6.3202247191.docx]

**DB69_length_356_mean_cov_6.3202247191**

```
GAATACATTAAAACACAAAGATACACACACACACACACACACACACACACACCTATCAACAGGTGAACATGGAATATCAGGTTGAAAAT
 >chr9:82616554-82616787 + E=6e-120
TAGCAAAGACCCCCATGTGCACACACACATTCACACCACTCATATTTAAGGAAAATGAATACCATAAAATAGAAAGTAGTATAGTAAAC

TTAAAATAAATATAACTCATATACATAGATAAAAATAAAGCATATTTCGTCAGGAGGAA|TGAGAACATGCGGTGTTTGGTTTTTTGTC
                                                             >chr10:96514839-96514958 +
CCTGCGATAGTTTGCTGAGAATGATGGTTTCCAGTTTCATCCATGTCTCTACAAAGGACATGAACTCATCATTTTTTATGGCTGCATAG
E=3e-57
T
```
